# Neurobiological Mechanisms Behind the Spatiotemporal Illusions of Awareness Used for Advocating Prediction or Postdiction

**DOI:** 10.3389/fpsyg.2012.00593

**Published:** 2013-01-04

**Authors:** Talis Bachmann

**Affiliations:** ^1^Laboratory of Cognitive Neuroscience, Institute of Public Law, University of Tartu (Tallinn branch)Tartu, Estonia

**Keywords:** consciousness, awareness, illusion, prediction, postdiction, timing, brain

## Abstract

The fact that it takes time for the brain to process information from the changing environment underlies many experimental phenomena of awareness of spatiotemporal events, including a number of astonishing illusions. These phenomena have been explained from the predictive and postdictive theoretical perspectives. Here I describe the most extensively studied phenomena in order to see how well the two perspectives can explain them. Next, the neurobiological perceptual retouch mechanism of producing stimulation awareness is characterized and its work in causing the listed illusions is described. A perspective on how brain mechanisms of conscious perception produce the phenomena supportive of the postdictive view is presented in this article. At the same time, some of the phenomena cannot be explained by the traditional postdictive account, but can be interpreted from the perceptual retouch theory perspective.

## Introduction

In the changing environment our brains inevitably provide us with a bit outdated percepts because of the time it takes to process new information (Eagleman, 2008; Nijhawan, 2008; Nijhawan and Khurana, 2010; Yamada et al., 2012). Obviously, this state of affairs is adaptively disadvantageous. Evolution must have provided us with some means to compensate or correct the often non-veridical perception vis-à-vis the actual appearance of the changing scene in order to enable subjects to act efficiently and interpret world around us veridically. The two most popular solutions for explaining how sensory-perceptual and sensorimotor systems may overcome, reduce, or re-interpret this processing delay dependent perceptual non-veridicality are prediction (Nijhawan, 1994, 2008; Kerzel, 2003; Cardoso-Leite et al., 2010 – all these associated with an increasingly popular Bayesian account of predictive encoding, e.g., Kersten et al., 2004; Bar, 2007; Hohwy et al., 2008) and postdiction (Eagleman and Sejnowski, 2000; Choi and Scholl, 2006; Eagleman, 2008; Buehner and Humphreys, 2010; Kawabe, 2011, 2012). (Approaches combining these accounts can be also acknowledged, e.g., Soga et al., 2009.)

The empirical evidence where the limits of the perceptual system in coping with challenges of the environmental stimulation come to the fore is surprisingly rich, consisting in many well established experimental awareness phenomena. In a recent review (Bachmann et al., 2011) the following examples are listed where spatiotemporal information is represented either non-veridically, surprisingly poorly or as if seeing more than is there: anorthoscopic perception, anthropomorphic perception effect of causality, attentional blink, Aubert–Fleischl effect, autokinetic effect, biological motion (Johansson effect), Cai and Schlag effect, change blindness, Cohene and Bechtoldt effect, color-phi phenomenon, cutaneous rabbit phenomenon, Czermak effect, feature attribution, feature inheritance, filled-duration illusion, flash-lag effect (FLE), (continuous) flash-suppression effect, flicker fusion, Fröhlich effect, Galli effect, induced-motion effect, Lawrence effect, line motion illusion (Hikosaka effect), masking effects, motion capture, motion induced blindness (MIB), Motoyoshi effect, multiple flash effects, path-guided motion, perceptual asynchrony effect, perceptual latency priming, phenomenal causality (Michotte) effect, proactive contrast facilitation, Pulfrich effect, repetition blindness, representational momentum, repulsion effects, sequential blanking, size transformation effects, sound-induced illusory flash phenomenon, standing wave illusion of invisibility, Stoper and Mansfield effect, stroboscopic motion, tandem effect, temporal context effect of brightness, temporal order reversal effect, Ternus–Pikler effect, tunnel effect, ventriloquist effect, voluntary-action effect on perception timing, wagon-wheel illusion, Zöllner effect. Many of these phenomena are used for providing evidence for predictive or postdictive accounts of explicit perception. In this paper I will focus on some of these phenomena, indicate whether the predictive or the postdictive account is consistent with them and describe how the action of the perceptual retouch theory based awareness mechanism explains these phenomena. The choice of the seven phenomena for the purposes of the present article is not haphazard. First, from the long list presented above only a couple of the phenomena have been frequently used in the context of experimentation and theorizing trying to test or juxtapose both the predictive and postdictive accounts of spatiotemporal processing. Thus, only for a relatively limited set of the phenomena there is a sufficiently voluminous published record of discussion relevant to our topic. Second, space would not permit a systematic analysis of all the phenomena in the context of prediction, postdiction, and the perceptual retouch theory. Third, in order to be able to compare the validity of the alternative theoretical accounts in explaining the phenomena, both of these accounts should have statements and working principles specific enough with regard to the spatiotemporal characteristics of the phenomena. This is in order to make the comparative evaluation possible. This also restricted our choice.

## Experimental Awareness Phenomena and the Two Accounts

The most studied and discussed phenomena we use in this paper can be listed as follows.

1. *Flash-lag effect* where a *moving* and a static (flashed) stimulus are compared for their relative position (e.g., Nijhawan, 1994). In perceptual awareness the moving object appears ahead of the static flashed object even though actually they are aligned when the flash is presented. The predictive account (Nijhawan, 1994, 2008) explains this phenomenon as a result of an active transformation of the percept of the moving stimulus according to this algorithm: compute the vector of the preceding position change → execute a corrective transformation of the ongoing percept formation (predictive encoding) → arrive at an illusory percept corresponding to a highly likely anticipated reality where the position of the moving object is shifted forward along the motion vector. Compared to the perceived position of the static flash this spatial advancement provides the foundation for the illusory flash-lag. However, this account cannot explain FLE found in the following experimental conditions: (i) the flash-initiated conditions where the moving stimulus begins to move only after the flash (i.e., no prediction basis is present; Khurana and Nijhawan, 1995), (ii) motion-reversing (Whitney and Murakami, 1998) conditions where the moving stimulus changes its motion direction, (iii) conditions where in addition to the moving reference stimulus another stimulus approaches the reference from an opposite direction and thereby provides a conflicting, canceling motion vector before the flash (Bachmann et al., 2012), (iv) conditions where the flash itself is also briefly in motion (Bachmann et al., 2003). Thus, the events after the flash must be responsible for the FLE. The postdictive account (Eagleman and Sejnowski, 2000; Eagleman, 2008) has an advantage here.
2. The FLE when a *spatially localized stimulation stream* changes its feature value (e.g., hue) and is evaluated against a flashed probe stimulus that has an invariant feature value (e.g., Sheth et al., 2000). Analogously to the motion version of the FLE, the perceived feature value of the flashed stimulus appears to lag behind the already seemingly advanced feature value of the changing stimulation. Prediction of the change does not work as an explanation here either. For example, when a target stimulus is presented in a stream of featurally invariant and spatially overlapping foil stimuli it is nevertheless perceived as appearing before the reference stimulus flashed in a neighboring position simultaneously with the target (Bachmann and Põder, 2001). Thus, neither motion nor feature change is decisive for the FLE. Prediction is useless, but some process that makes a newly appearing stimulus slower in terms of its delay to consciousness compared to an in-stream stimulus must be responsible for the effect. The postdictive account (Eagleman and Sejnowski, 2000; Eagleman, 2008) has explained the FLE like this: encoding of the features of the changing object/event → waiting for the slowest feature to have been encoded → re-interpretation of the encoded signals post-dicted back in time to the moment of flash to compensate the inevitable delay in feature processing. However, because in Bachmann and Põder (2001) FLE was found also when the target flashed in the stream and the reference flash presented out of the stream were simultaneous and identical, but different from the stream items, it is difficult to understand how the postdiction could lead to the FLE illusion.
3. *Perception of causality* in the “colliding stimuli” displays (e.g., Choi and Scholl, 2006). It has been found that visual events can determine whether a collision is perceived in an ambiguous situation even when those events occur after the moment of “impact” in the putative collision has already passed. This is consistent with the postdictive account of perception. Here conscious perception again appears not as an instantaneous percept formation, but comes about by integrating information presented within short temporal windows, so that new information can influence the immediate past in conscious awareness.
4. The *Fröhlich effect* where the first perceived location of a newly appearing moving object is shifted forward along the motion direction (e.g., Müsseler and Aschersleben, 1998). This effect is consistent with what the predictive account (Nijhawan, 2008) would expect, provided that the computation of the motion signals is carried out very fast. However, the postdictive account seems in trouble here. It remains unexplained why – even if post-dictively and in retrospect – the first positions of the moving object remain out of awareness.
5. *Representational momentum* – an effect where the perceived end-position of a moving object when it stops “overshoots” its actual position (e.g., Müsseler et al., 2002). This phenomenon again is well accounted for by the predictive theory, but not so easily by the postdiction theory.
6. *Reappearance* in awareness of the stimuli made subliminal *in the MIB* displays (e.g., Mitroff and Scholl, 2004; Kawabe et al., 2007). When the stimulus that disappeared from awareness a moment ago changes its physical appearance when subjectively invisible (e.g., is slanted during its blind episode), is switched off when invisible and reappears in awareness again later, the consciously perceived quality of it (e.g., slant) corresponds not to how it appeared when last in awareness, but represents the stimulus how it was presented subliminally before disappearance. This is a bizarre mix of postdiction and prediction accounts of conscious perception because a former stimulus state is reinstated (caused by flash or switch-off), but also its perceptual characteristics were retrospectively reinstated in the direction of change that was indicated unconsciously. The most intriguing work in this paradigm was presented by Wu et al. (2009). They showed that a flash that caused reappearance of the target stimulus in awareness (after having been “subliminated” by MIB) was itself consciously perceived as appearing later than the reappearing target. At first this may seem paradoxical because the cause is perceived after its effect. Here, two important insights to our knowledge about the neural basis of conscious experience have to be noted. First, the findings by Wu et al. (2009) suggest that before reaching consciousness, the non-conscious representation has to be processed for about 100 ms. Second, these results show that there has to be a non-specific mechanism which brings the non-conscious specific representation to consciousness – any explanation for these results requires a process that is activated by the flash but at the same time acts on the representation of the target (and is, therefore, not specific for the target). I will return to this theme in the next part of this article. As for the predictive account there is nothing supportive in the Wu et al. (2009) data. Predicting the past before future is suspect. The postdictive account is better (i.e., reinstates what was there earlier, albeit in the pre-conscious format), but requires additional assumptions for explaining why the flashed stimulus that caused the reappearance of the target in awareness was not perceived at the same time with the reappeared target. According to Eagleman (2008), for the visual brain to correctly align the timing of events in the world, it may have to wait about 100 ms for the slowest information to arrive – thereby allowing the visual system to discount different delays imposed by the early stages. In the Wu et al. (2009) experiment the flash comes when also the target stimulus *is* present and *has been* present. Thus postdicting the flash-plus-target event back should have anyway represented both the flash-stimulus and the target stimulus together.
7. *Anorthoscopic perception* where the full shape of a moving stimulus is perceived despite that only part of its contours are visible through a slit at any moment in time is another phenomenon relevant in our context (e.g., Zöllner, 1862; McCloskey and Watkins, 1978; Aydin et al., 2008). Because this happens also with new stimuli, the shape of which is unknown beforehand to the perceivers, the prediction account cannot explain this effect. If the system does not know the regularities of change on which to found its predictive transformation, this kind of transformation is not possible. However, the postdictive account assuming a time consuming spatiotemporal integration of the unpredictable shape signals and motion signals after they have been processed can explain the shape formation post factum. Due to the space limitations I will skip here some other relevant phenomena for which there is sufficient level of specification allowing comparison with our theories such as the *line motion illusion* (Hikosaka et al., 1993) or the *Tandem Effect* (Müsseler and Neumann, 1992). Suffice it to say that the explanations for them are basically similar to what will be given in the next section of this article.

We saw that predictive and postdictive theory both had their successes in explaining the listed phenomena. At the same time these phenomena are the cases where subjective, conscious-awareness-level representation is inconsistent with the objective, physical characteristics of the presented stimulation. How the known properties of the brain mechanisms necessary for contentful conscious perception may be causally relevant in leading to these illusory phenomena? Because these phenomena are typically the empirical basis for the theoretical arguments either in favor of the prediction or the postdiction account it is useful to see whether the workings of the awareness mechanism provide explanations for the phenomena and thus provide a mechanistic basis for either one of the theoretical accounts.

## The Mechanisms for Perceptual Awareness Vis-à-Vis the Phenomena

**Figure 1:**
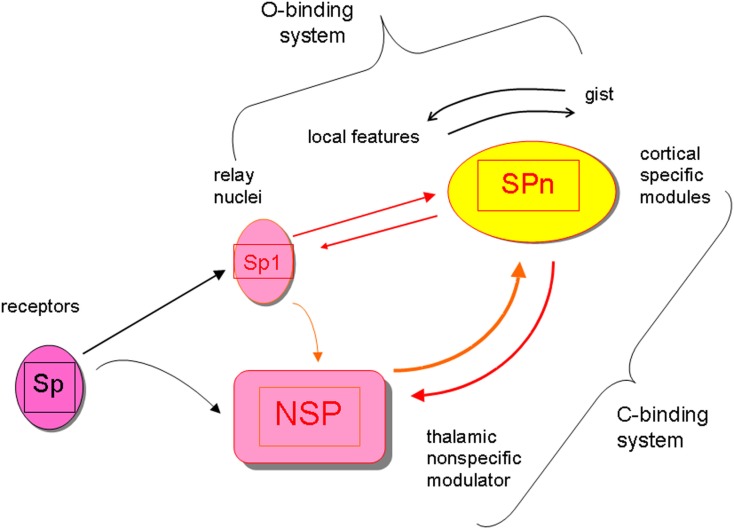
**The general framework of the perceptual processing system featuring interaction of content-specific channels and modules (Sp, Sp1, SPn) and the non-specific system of modulation with its core in the subcortical nodes (NSP)**. The specific system for contents works fast, can work pre-consciously, it integrates objects from features and events from objects (it is the O-binding system). Contents are represented by the cortical SPn. The non-specific system of modulation works slowly (ignition of the boost in its activity by a stimulus takes longer time), requires interaction between cortex and subcortex, but it is necessary for modulating the activity of SPn up to the level or activity mode where awareness of the contents represented by SPn emerges. (The interactive NSP + SPn system is the C-binding system subserving the function of binding the pre-conscious SP-representations with the global scene represented in the conscious awareness format.) Because modulation of SPn by afferents from NSP takes longer time than pre-conscious encoding of SPn contents from Sp-Sp1, any stimulus-input (e.g., Si) has a shorter latency to reach awareness when preceded by some other stimulus-input (e.g., Si-1) compared to when no preceding stimulation is presented and Si is presented alone. If Si is presented alone, but changes its attributes when pre-conscious, it may emerge in conscious awareness in the already changed capacity (e.g., shifted location, changed color, transformed shape).

In this article I stick to the neurobiological mechanisms responsible for producing consciousness-level perceptual awareness as was suggested in the perceptual retouch theory (Bachmann, 1984, 1994, 2007). Consciousness-level visual perception of the environmental objects involves two types of binding operations, which both require some time to be carried out. First, there is the content-specific binding of features to integrated objects which is accomplished by the selectively tuned cortical stimulus-specific (SP-) modules in V1, V2, V3, V4, V5, and various temporal lobe areas (Koch, 2004; Rose, 2006; Gazzaniga, 2009). The processing by the SP-system can be carried out pre-consciously, without a concomitant awareness (explicit perception) of the presented, encoded and featurally bound stimuli (Naccache and Dehaene, 2001; Ruz et al., 2003; Kotchubey, 2005; Dehaene and Changeaux, 2011; van Gaal et al., 2011). Secondly, awareness of any of these object requires the binding of the neural representation formed by SP-operations with the more global and non-specific neural activity supported by the thalamo-cortical processes of neuromodulation (Bachmann, 1994; Purpura and Schiff, 1997; Ribary, 2005; Bogen, 2007; Alkire et al., 2008; Urbano et al., 2012) that I label as NSP (for “non-specific”). The NSP-processes do not communicate specific contents of the environmental stimuli, but they are necessary in order to bring the specific contents represented by SP-processes into consciousness-level representation. So, paradoxically, *non*-specific is specific for providing the phenomenal capacity for the specific contents. Interaction between cortical SP-modules and the subcortical (e.g., non-specific thalamic) nuclei constitutes the key mechanism for modulation of the SP-carried perceptual contents by the NSP. The boost of NSP-activity is caused by the presented stimulation and especially notably by the appearance of the new inputs. (The ignition of the NSP system is one of the subparts of the orienting reflex circuitry, its early working part.) Importantly, the receptive fields of the neurons constituting NSP are larger than the receptive fields of the neurons in the cortical SP whose function is to process specific incoming signals from the presented stimuli. Therefore, presentation of a certain specific stimulus with its specific content K can ignite a NSP-process which is capable of modulating the activity of some other neurons X with different specific content (even before the signals for X have been presented). The presynaptic inputs from both, SP-channels (from receptors via the lateral geniculate body up to the cortex) and NSP-channels (from the thalamo-cortical modulation system) converge on the cortical SP and both types of inputs regulate the excitatory postsynaptic potentials of the SP neurons. When this presynaptic input combining somatic and dendritic presynaptic effects from direct SP-channels and indirect NSP-channels is strong enough (e.g., as applied onto pyramidal neurons with their characteristic long apical dendrites), the specific neurons begin firing or increase their firing rate. When only SP-channels are active for representing actual stimulus objects but dissociated from NSP influence, no consciousness of the perceptual contents of these objects can be experienced (Bachmann, 1994; Koch, 2004; Ribary, 2005; Bogen, 2007). The SP works faster than NSP which means that pre-conscious perceptual representation is formed ahead in time with regard to the time when NSP-modulated contents become consciously available. (The time difference between an effective pre-conscious SP-encoding of objects with their bound features and an effective process of NSP-modulation necessary for awareness to emerge amounts to about 50–150 ms.) Figure 1 summarizes the general framework of the SP + NSP processing system.

Within this framework, the phenomena reported in the current article can be explained as follows.

1. Flash-lag effect where a moving and a static (flashed) stimulus are compared for their relative position and the flash appears to lag behind (e.g., Nijhawan, 1994). The retouch theory explanation (Bachmann et al., 2003, 2012; Bachmann, 2010) is this: because the action of NSP takes more time than SP-encoding and because no awareness of the SP-represented contents emerges before NSP-modulation has had its effect, the percept in awareness emphasizes features that are or become present in SP somewhat later. For the features of the static flash this means that its initial position as stored in sensory memory will be “retouched” for consciousness, but for the features of the moving stimulus this means “retouching” an advanced spatial position for consciousness. (Additionally, the lingering sensory trace of the moving stimulus is erased for SP by a Reichardt type of movement detector; Reichardt, 1961.) This creates the illusion of a spatial lag. This explanation is valid also for the flash-initiated conditions, the conditions where the post-flash movement directions are unpredictable and the conditions where the pre-flash stimulation includes contradictory motion direction signals that could nullify or complicate prediction (Khurana and Nijhawan, 1995; Whitney and Murakami, 1998; Bachmann et al., 2012). In some sense the retouch theory explanation can be considered as a variety of the latency difference account. For example, Whitney and Murakami, 1998, p. 657) state that “The simplest explanation is that the neural delays for the flash and the moving bar are different … approximately 45 ms … represents the difference between the latencies for moving and flashed stimuli. Specifically, the delay for the moving bar is shorter…, perhaps because responses of motion detectors at one location facilitate the response of other detectors along the expected path of motion.” Actually, there are some important differences between the simple latency difference account and the retouch theory explanation. It is not essential that processing of the *motion* signals may be faster, but that any signals with precedence have shorter delay to arrive awareness because the action of the NSP (a system necessary for awareness of the already pre-consciously represented stimuli) has been activated in advance and the signals later in-stream win time to reach conscious awareness. The latency difference means latency-to-awareness, difference. Furthermore, as we will see subsequently, the retouch mechanism explains FLE also in the conditions where motion is not the case and static stimuli are presented. The retouch mechanism supports the postdictive account, but it also does not need the somewhat mystical “referral back in time” (Eagleman and Sejnowski, 2000).
2. Now let us consider the FLE effect when a spatially localized stimulation stream changes its feature value (e.g., hue) and is evaluated against a flashed probe stimulus that has an invariant feature value (e.g., Sheth et al., 2000). FLE in this case is produced similarly to what was described in item 1 above. For the newly appearing reference stimulus the fast SP-process and a slow NSP-process are evoked. When the slow NSP-modulation becomes effective, it helps to actualize the former feature value of the reference stimulus stored in sensory memory. For the object features within the stream of the changing stimulation the process is relatively faster because the former stimulation-instances from the stream have ignited the NSP-process ahead in time and upon arrival of the new signals from the subsequent input within the stream the corresponding feature values become available for awareness relatively earlier. Notice that an analogous flash-lag (i.e., in-stream facilitation) effect was obtained when the target stimulus within the stream was a feature singleton and preceding items in-stream did not carry any predictive cues (Bachmann and Põder, 2001). Importantly, this stresses the non-specificity of the mechanism that modulates SP-data for awareness. Any localized or spatially nearby input can ignite NSP-modulation for subsequent stimuli that can take advantage of this process even if featurally the stimuli are considerably different. Thus, the predictive account cannot help here. Surprisingly, the postdiction account also has its difficulty here. The FLE is present also when the in-stream target and the out-of-stream reference are presented simultaneously and referral back in time after the resetting of the timer due to the flash (Eagleman and Sejnowski, 2000) should find simultaneous identical stimuli – one in the stream and the other out of the stream. (Notice that spatial positions of the stimuli do not change and the in-stream target and reference are feature wise identical stimuli.)
3. Perception of causality in “colliding stimuli” displays (e.g., Choi and Scholl, 2006) where visual events can determine whether a collision is perceived in an ambiguous situation even when those events occur after the moment of “impact” in the putative collision has already passed: due to the slowness of the NSP action an explanation similar to the FLE can be put forward from within the retouch theory context. The general postdictive account seems valid here.
4. The Fröhlich effect where the first perceived location of the moving object that moves out from behind the occluder is shifted forward along the motion direction (e.g., Müsseler and Aschersleben, 1998). This effect is consistent with what the predictive account (Nijhawan, 2008) would expect, provided that the computation of the motion signals is carried out very fast. The postdictive account cannot explain why – even though post-dictively and in retrospect – the first positions of the moving object remain out of awareness. The retouch theory naturally explains the Fröhlich effect: the slow NSP-modulation arrives at the active SP-representation of the moving stimulus when its position has been advanced during this NSP-delay. (Why the former positions of the moving stimulus remain invisible can be explained by the Reichardt detector effect canceling the trailing edge of the moving stimulus sensory trace within SP.)
5. The next relevant phenomenon is representational momentum where the last perceived position of a moving object when it stops “overshoots” its actual position (e.g., Müsseler et al., 2002). Predictive account is valid here, but both the postdictive general account and the perceptual retouch mechanism based explanation cannot provide any good solution unless accepting that the SP-contents may be influenced by the mechanisms that carry out extrapolatory correction.
6. Reappearance in awareness of the stimuli made subliminal in the MIB displays (e.g., Mitroff and Scholl, 2004; Kawabe et al., 2007) is our next phenomenon. The MIB stimulation condition (the moving background noise and the static target stimuli being in conflict) causes decoupling of the NSP influences selectively from the SP-representation of the target. Because SP works pre-consciously and NSP is the necessary, albeit slow modulation mechanism for conscious awareness of the SP-contents, the following occurs. When NSP reassumes its effective work at a later moment (either spontaneously or due to an on- or off-transient) it also retouches the – possibly changed – feature values of the target. The paradoxical appearance in awareness of the effect-related target stimulus before the cause-related flashing stimulus (Wu et al., 2009) can be explained by the perceptual retouch mechanism. Bachmann and Aru (2009) suggested that “When the target object such as used in the/Wu et al., 2009/fades from awareness, the SP remains active in the mode sufficient for the representation of the specific contents for the target, but has become dissociated (i.e., desynchronized) from the NSP-activity necessary for consciousness of the target. When the flashed object is presented, two processes are triggered – the SP-process for representation of the contents of the flashed stimulus and the (boost of or perturbation in the) NSP-process. This facilitated (or reset) NSP-activity leads to binding of the already present pre-conscious SP-activity of the target with global consciousness-level representation. This binding process takes little time because there is no need for build-up of the content-specific neural representation for the target. In a putative computational model, only phase resetting between the already functioning two oscillatory activities is required. Target reappears in consciousness fast. However, the flashed object appears in consciousness later because the corresponding SP-representation of the flash has to be built up ab ovo from the lower levels up to the higher pattern levels and this takes some time. Therefore, the NSP that brings contents to awareness finds the SP-contents of the target ready on the “waiting list”; however, this NSP-activity has to wait until the SP-contents of the flashed object become ready (i.e., bound to the object representation to be bound into consciousness). The predictive account is not useful here because predicting the past before future is suspect. The general postdictive account needs some ways to explain why the flashed stimulus that caused the reappearance of the target in awareness was not perceived at the same time with the reappeared target. Postdicting the flash-plus-target, event back should have anyway represented both – the flash-stimulus and the target stimulus.
7. The last phenomenon we consider is anorthoscopic perception where the shape of a moving stimulus is perceived, although only part of its contours are visible through a slit at any one moment (e.g., Zöllner, 1862; McCloskey and Watkins, 1978). As this happens also with new stimuli unknown to observers, the prediction account cannot explain this effect. The postdictive account assuming a time consuming spatiotemporal integration of the unpredictable shape signals and motion signals after they have been processed can explain the shape formation post factum. Perceptual retouch account in its present form cannot explain the effect unless the NSP effects can be very slow and the SP-modules are termed to include high-level visual-cognitive representations enabling more complex dynamic transformations.

Table 1 summarizes my evaluations of whether the predictive account, postdictive general account, and the retouch mechanism based mechanistic explanation are consistent with the seven spatiotemporal phenomena of awareness used here for our analysis.

**Table 1:** **Evaluation of the consistency of the three theoretical explanations for the seven spatiotemporal perceptual awareness phenomena**.

The phenomenon (see text)	Predictive account	Postdictive general account	Perceptual retouch mechanistic explanation
1	+/−	+	+
2	+/−	+/−	+
3	+	+	+
4	+	−	+
5	+	−	−
6	−	+/−	+
7	−	+	−
Sum	4.0	4.0	5.0

*+: Account/theory and the phenomenon are consistent*.

*−: Account/theory and the phenomenon are not consistent*.

*+/−: Consistency satisfied depending on which variety of the phenomenon is used*.

It is easy to see that the predictive as well as postdictive account both can explain more than half of the phenomena under consideration. However, the distribution of the consistency ratings is different. Except for the perception of causality in collision which can be explained by both accounts without reservations, the other phenomena are more puzzling for either the prediction or the postdiction theory or both. Certain special varieties of motion-involving FLEs and static FLEs cannot be accounted for by these theories. Moreover, while the phenomena involving a kind of inertia effects (Fröhlich effect and representational momentum) are well accounted for by the predictive account, they cannot be easily explained by the postdictive account. On the other hand, the predictive theory is in trouble trying to explain reappearance in awareness after MIB and the anorthoscopic perception, both of which can be either fully or partly explained by postdiction. In the majority of cases the retouch mechanism also explains the phenomena and where it does, it does this without reservations (phenomena 1, 2, 3, 4, 6 from Table 1). For the “overshoot” effect in the representational momentum phenomenon and for the “creative” formation of the full shape from its dynamic fragments in the anorthoscopic effect the retouch theory does not have any specialized modules helping to lead to these effects (items 5 and 7 in the Table).

From the Table 1 and the above analysis we see that no theory is able to explain the effects singlehandedly. Each one has its advantages and disadvantages. For some phenomena, the accounts are not exclusive in their explanations and can be mutually consistent. For example, the perceptual retouch mechanism can be considered as the neurobiological mechanism by which the phenomena are produced, which in turn may become subject for the interpretational higher order cognitive mechanisms working according to the abstract principles of postdiction (1, 2, 3, 6 in the Table). Regarding some phenomena, the contributions of the mechanisms suggested in the three theories may be additive, such as when motion extrapolation in certain varieties of the FLE or causality-from-collision experimental setups are used as examples (items 1–3 in the Table). Importantly, future experiments must be useful in trying to disentangle these relative contributions by clever experimental designs allowing control over the variables specific to each of the theories.

The general picture as it emerges from this analysis reveals some main differences between the theoretical accounts. The predictive account may be relatively restricted to the lower level effects involving motion and simple feature change analysis. The postdictive account fares better with effects where relatively high-level visual-cognitive processes play their part. The perceptual retouch theory completes the picture by providing the neurobiological foundations for the effects where conscious perception represents the dynamic environment non-veridically because the NSP component of the retouch mechanism is slow. As the NSP component is necessary for upgrading the already processed information for conscious awareness, the slowness dependent illusions are inevitable in the direct perception. In the regulation of behavior and cementing general knowledge of the dynamic world around the subject higher level cognitive mechanisms implied in the postdiction account may be of help.

Our comparative analysis suggests that a uniform explanation of all of the observed effects seems impossible right now. There is a complex interacting set of low-level and high-level mechanisms and also the capacity of the visual system to execute sufficiently sophisticated computations and encodings unconsciously. Given the variability and complexity of the spatiotemporal stimulation a subject may encounter and lack of unequivocally interpretable and invariant set of cues to be processed, a single one relatively simple mechanism may not be sufficient to account for all possible perceptual effects. Though having said this, it is surprising that the perceptual retouch mechanism can explain majority of the phenomena without reservations.

## Concluding Remarks

In this paper I presented a mechanistic explanation for the typical visual awareness phenomena that have been used for testing and advancing predictive and/or postdictive accounts of conscious perception. It seemed natural to look for the mechanisms precisely there where neurobiological data has shown what are the necessary brain processes for the emergence of a contentful perceptual experience (Bachmann, 1994; Koch, 2004; Ribary, 2005; Bogen, 2007). This small endeavor showed that both the predictive account and the postdictive traditional account can explain more than half of the “litmus-test” phenomena typically used in visual awareness studies in the present theoretical context. Surprisingly or not, the perceptual retouch theory based mechanistic explanation produced even a bit higher summary rating for the consistency (see Table 1). This explanation also supports several of the postdictive account principles, however this is without the need to invoke a somewhat mystical concept of referral back in time. Simply the delay to conscious awareness of featured perceptual information depends on whether the target stimuli were preceded by other input signals from spatially close/overlapping locations or not. If there was precedence, the NSP-processes are prepared to have their effect ahead in time and subsequent stimuli reach awareness relatively faster.

I do hope also that the perspective suggested here and based on the perceptual retouch theory of conscious perception might be useful in order to specify the so-called postdictive account more precisely in terms of the underlying neural mechanisms. Ultimately, it may turn out that postdiction in its radical sense may not be needed at all. On the other hand, the predictive account also cannot be sufficient. Not least because there are too many experimental effects of conscious vision unaccountable by the traditional approaches.

## Conflict of Interest Statement

The author declares that the research was conducted in the absence of any commercial or financial relationships that could be construed as a potential conflict of interest.
